# Silent teachers: Narratives from the simulation lab

**DOI:** 10.1111/medu.15711

**Published:** 2025-05-20

**Authors:** Aaron Lawson McLean, Anna C. Lawson McLean

**Affiliations:** ^1^ Department of Neurosurgery Jena University Hospital – Friedrich Schiller University Jena Jena Germany

## Abstract

**Introduction:**

Patient mannequins are ubiquitous in simulation‐based medical education, yet their own ‘stories’ remain untold. This paper adopts a playful, phenomenological lens, amplified by artificial intelligence (AI), to explore the fictional ‘lived experiences’ of these silent teachers and reflect on their pedagogical significance.

**Methods:**

Fictional narratives were generated for three representative mannequins – Mr. Heart Attack, Mrs. Difficult Birth and a composite ‘Geriatric Triplet’ representing common geriatric syndromes – using a large language model (ChatGPT 4.0). These narratives were analysed using qualitative methods (narrative inquiry, phenomenology, content analysis) to explore their ‘experiences’ and pedagogical roles.

**Results:**

The AI‐generated narratives depicted mannequins possessing distinct ‘personalities’ and reflections on their roles (e.g., Mr. Heart Attack's resignation, the Triplets' patience). They highlighted key learning opportunities, such as managing clinical uncertainty (Mr. Heart Attack), holistic care (Triplets) and navigating high‐stakes emotional scenarios (Mrs. Difficult Birth). Themes emerged around clinical decision‐making, teamwork, communication and empathy.

**Conclusion:**

This playful yet reflective study highlights mannequins as more than inert tools, using their AI‐generated ‘voices’ to underscore key simulation principles. It also demonstrates a novel methodological approach, prompting reflection on narrative, perspective and the potential roles of AI in shaping educational discourse.

## Abstract 2

If mannequins could speak, what stories would they tell? This playful exploration uses AI‐generated narratives to reflect on empathy, teamwork & hidden lessons in simulation training. #MedEducUnleashed

## 1 INTRODUCTION

It is a truth universally acknowledged that a medical student in possession of a stethoscope must be in want of a patient. But in the dynamic theatre of medical education, learning extends beyond interactions with human patients. Enter the unsung co‐stars: the patient mannequins. These silent mentors, ubiquitous fixtures in simulation labs worldwide, play crucial roles in honing the skills of future healthcare professionals.^1^, ^2^, ^3^, ^4^ While often viewed simply as tools, they are central figures in countless simulated dramas.^5^, ^6^ Akin perhaps less to the Hamlets and more to the Rosencrantzes and Guildensterns of the educational stage – supporting characters whose perspectives enrich the main narrative – they have perspectives that often go unheard. Our exploration follows a literary tradition of delving into the overlooked stories residing in the margins, offering a touch of Nietzschean merriment along the way.

What might we learn if we could access their ‘experiences’? Despite the invaluable role mannequins play, their ‘subjective’ contributions remain largely unexplored in the literature.^7^, ^8^, ^9^, ^10^, ^11^ Adopting a playful phenomenological lens, amplified by the narrative capabilities of artificial intelligence (AI), we aim to imagine the world through their unmoving eyes. This paper takes a whimsical premise – giving voice to mannequins via AI – and treats the resulting narratives seriously as textual data, using established qualitative methods to explore what insights might emerge regarding simulation practice and pedagogy. By attempting to uncover the ‘untold stories’ of these silent instructors, we seek to spark fresh reflection on their role in fostering clinical skills, empathy and critical thinking, blurring the lines between the simulated and the real in provocative ways.

## 2 METHODS

### 2.1 Conceptual approach

Our venture into the ‘psyche’ of mannequins employed a hybrid methodology. Narrative inquiry focuses on understanding experiences as told through stories; here, we used AI to *generate* the stories before analysing them. A phenomenological lens was adopted not to access genuine consciousness (an impossibility), but as an *interpretive stance*, aiming to understand the ‘what it is like’ depicted within the AI‐generated texts, temporarily bracketing our usual biomedical view of these objects.^12^, ^13^, ^14^

### 2.2 Participants’ and narrative generation

We selected three archetypal mannequin roles common in simulation labs: ‘Mr. Heart Attack’ (acute care/resuscitation), ‘Mrs. Difficult Birth’ (obstetrics/emergency) and a composite character dubbed the ‘Geriatric Triplet’ embodying common geriatric syndromes (cognitive impairment, falls, polypharmacy). An advanced large language model (LLM), ChatGPT (version 4.0, OpenAI, San Francisco, CA, USA), was used to generate fictional first‐person narratives for each. Researchers provided iterative, conversational prompts designed to elicit distinct ‘personalities’ and reflections based on the mannequins' typical roles and interactions within simulation scenarios (see Supplementary Material for prompt details). Table 1 presents edited excerpts from these narratives to illustrate their flavour.

**TABLE 1:** Patient mannequin narratives (edited excerpts).

Narrative element	Mr. Heart Attack	The Geriatric Triplets (composite)	Mrs. Difficult Birth
**Core ‘Experience’**	“Beat, stop, jolt, repeat,” sighs Mr. Heart Attack. Decades as the vanguard for ACLS training. Chest rising under eager hands, punctuated by the defibrillator's fire. “I've died more times than I can count, yet here I am, ready for another round.”	Representing cognitive impairment, falls, polypharmacy. Narrative laced with wisdom, patience. Nudging learners towards holistic care. Chuckles at bewildered students managing 10 drugs or nuanced dementia evaluations. “They learn, slowly, that it's not just about the disease, but the person living with it.”	Stories of tension, urgency, relief. Every possible birth scenario, from routine to shoulder dystocia. Newborn's ‘wail’ signals success or complication. Students' frantic manoeuvres testament to raw emotions. “The air crackles… learners navigate the storm I represent.”
**Pedagogical ‘Insight’**	Speaks volumes about clinical decision‐making, procedural skills, teamwork, communication. Reminisces about palpable tension before critical decisions, adrenaline surge of correct actions, impactful debriefings post‐‘resuscitation’. “They learn failure isn't final, but a step towards competence.”	Advocates for comprehensive geriatric assessment, preventive care, empathy, compassion. Underlines significance of interprofessional collaboration. “Reminding them that good care is never a solo performance but an orchestra requiring harmonious teamwork.”	Instills technical skills but also insight into patient communication, consent, dignity, respect through ‘simulated screams and silent cooperation’. Highlights gravity of empathy in high‐stakes scenarios. “Reminding learners that behind every clinical case is a human experience, deserving of compassion even amidst chaos.”

### 2.3 Analysis

These generated narratives, treated as qualitative data, were then subjected to an integrated qualitative analysis drawing on principles from narrative inquiry, interpretative phenomenology and thematic content analysis.^12^, ^13^, ^14^, ^15^ This involved: (1) repeated reading of the narratives to gain familiarity; (2) initial coding to identify key descriptive, linguistic and conceptual elements; (3) development of emergent themes related to the mannequins' ‘experiences,’ perceived roles and interactions; (4) interpretation of these themes through the phenomenological lens, focusing on the depiction of embodiment, temporality and intersubjectivity within the simulation context; (5) identifying recurring themes, including the emotional tones attributed to the mannequins within the generated text.

### 2.4 Rigour and reflexivity

Methodological rigour, within the playful constraints of this project, was sought through investigator triangulation, where members of the research team independently reviewed the narratives and analytical themes before reaching consensus through discussion. The appropriateness and coherence of the final themes were revisited against the original narratives. A reflexive approach was maintained throughout, acknowledging our dual roles as educators familiar with simulation and researchers interpreting novel AI‐generated text. We critically considered how our own experiences and assumptions might shape the interpretation of the mannequins' ‘perspectives’. We treated the analysis of the *text* seriously, applying the chosen analytical methods systematically to the generated data.

### 2.5 Epistemological and philosophical framework

Epistemologically, our approach blends elements. The LLM's generation process, based on patterns in its training data, has deterministic aspects. However, our prompting strategy and subsequent interpretive analysis introduce a strong interpretivist dimension, focused on deriving meaning from the generated text.

Philosophically, by employing an LLM to ‘speak for’ the mannequins, we adopt a post‐structuralist stance. This stance challenges the traditional view of the human author as the sole source of meaning, suggesting meaning arises through language and interaction. Here, the AI acts as a non‐human ‘author’, and our analysis explores the meanings emerging from this human‐AI interaction in the context of medical education. This stance encourages us to see how meaning can be co‐constructed and how non‐human elements contribute to the educational environment.

### 2.6 Ethical considerations

As the narratives are fictional and generated by AI based on archetypal roles rather than real individuals, traditional ethical concerns like patient privacy or consent are not applicable. The study was exempt from formal ethics board review based on institutional guidelines for non‐human/simulation‐based research.

## 3 RESULTS

The AI‐generated narratives provided distinct, imagined ‘voices’ for the three mannequin roles (Table 1).

### 3.1 Mr. heart attack

His narrative conveyed a sense of weary resignation (“Beat, stop, jolt, repeat”) coupled with a perceived purpose in the repetitive cycle of simulated cardiac arrest. The text highlighted the tension, critical decision‐making (“palpable tension… before a critical decision”) and teamwork inherent in resuscitation scenarios, as ‘observed’ from his inert perspective.

### 3.2 The geriatric triplets

This composite narrative emphasized patience and the complexity of multi‐morbidity (“slow‐paced, complex cases”). It portrayed the challenges learners face with polypharmacy and nuanced assessments, positioning the Triplets as advocates for holistic, person‐centred care and interprofessional collaboration (“good care is never a solo performance”).

### 3.3 Mrs. difficult birth

Her narrative focused on the emotional intensity and urgency of obstetric emergencies (“tension, urgency, and relief”). It depicted the high stakes, the blend of technical skill and communication required (“simulated screams and silent cooperation”), and the importance of empathy in emotionally charged situations (“behind every clinical case is a human experience”).

Across the narratives, several key themes emerged related to the simulation experience itself: the importance of clear communication and teamwork (Mr. Heart Attack, Triplets), managing uncertainty and decision‐making under pressure (Mr. Heart Attack, Mrs. Difficult Birth), the need for empathy and patient‐centredness (Triplets, Mrs. Difficult Birth) and the value of debriefing and learning from errors (implied in Mr. Heart Attack's repetitive ‘deaths’). The analysis also identified distinct emotional tones attributed to the mannequins by the AI (Table 2), adding another layer to their fictional ‘personalities’.

**TABLE 2:** The attributed emotional spectrum of mannequins (via AI narrative).

Mannequin	Atributed emotion	Illustrative narration extract (AI‐generated)
**Mr. Heart Attack**	Resigned Acceptance	“Another MI, another day… another opportunity to teach these hopefuls the ropes. I guess I should be proud, in a peculiar way.”
**The Geriatric Triplets**	Unending Patience	“Yes, dear, I know I'm Triplet #2… That's your cue, doc! Take your time, think it through.”
**Mrs. Difficult Birth**	Anticipatory Tension	“Oh, here we go again. The lights dim, the atmosphere grows tense… It's showtime!”

## 4 DISCUSSION

This study used a novel, AI‐driven approach to playfully explore the ‘inner lives’ of patient mannequins, aiming to generate fresh reflections on simulation‐based education. While the premise is playful, the analysis of the generated narratives prompts serious consideration of several aspects of simulation pedagogy and the learning environment.

### 4.1 Mannequins as more than tools

The narratives, imbued with personality and perspective by the AI, challenge us to view mannequins as more than just inert pieces of plastic and circuitry. Considering the mannequin's ‘viewpoint’, even as a thought experiment prompted by these texts, encourages a shift in perspective for both learners and educators. Might it subtly influence how educators design scenarios or facilitate debriefings, perhaps encouraging them to articulate the ‘patient's’ experience more vividly? Could encouraging learners to briefly ‘empathize’ with the mannequin – to consider the scenario from that focal point – enhance their engagement or sensitivity to the human factors involved? This approach primarily serves as a catalyst for dialogue about how we relate to and utilize these essential educational tools.^16^

### 4.2 Reinforcing simulation principles

On a more serious note, the themes emerging from the narratives resonate strongly with the core principles of effective simulation‐based education. The AI‐generated stories highlighted the importance of psychological safety (allowing learners to struggle and make mistakes, as Mr. Heart Attack ‘observes’), the necessity of structured debriefing (implied by the learning cycle), practicing teamwork and communication skills and developing clinical reasoning in complex situations.^17^ Mrs. Difficult Birth's narrative underscores the role of simulation in developing not just technical proficiency but also affective skills like empathy and managing stress under pressure.^18^ The mannequin narratives, therefore, serve as unconventional reminders of established best practices in the field.

### 4.3 Methodological reflections and post‐humanist whispers

The *process* of using an LLM to generate narratives proved effective in creating rich textual data for analysis. This methodological approach itself prompts reflection on how technology might be used to foster creative explorations and discussions within medical education, moving beyond purely technical applications.^19^, ^20^ This playful exploration also aligns with post‐humanist perspectives, which decenter the human and examine the roles of non‐human elements in shaping experiences and knowledge.^21^ It invites us to consider mannequins not merely as passive tools but as ‘actants’ within the educational network. Their ‘performance’ (enacting clinical scenarios), alongside the AI's generative role, challenges anthropocentric notions of teaching and learning, hinting at distributed cognition and learning assemblages where human and non‐human elements interact dynamically.^22^

### 4.4 Signalling seriousness

While the study embraces whimsy, its underlying purpose is to provoke reflection on established practices and future possibilities in simulation. The use of familiar qualitative methodologies applied to unconventional ‘data’ serves as a methodological exercise, demonstrating how these techniques can be adapted to explore novel questions, even humorous ones. Turning to the implications, the study encourages educators to continually reflect on how simulation tools are deployed and perceived, and how the design of the learning environment shapes learner experience.

### 4.5 Limitations

This study has several limitations inherent in its playful design. The narratives are AI‐generated fictions, not authentic experiences and reflect the biases and patterns within the LLM's training data and the researchers' prompts.^23^, ^24^ Our selection of only three mannequin archetypes limits the diversity of ‘perspectives’. Furthermore, the AI‐generated narratives, influenced by the prompts provided and the underlying data the LLM was trained on, may inadvertently reflect or even reinforce existing biases or stereotypes prevalent in healthcare and simulation (e.g., the ‘difficult birth’ scenario potentially lacking cultural nuance).^25^ Future work could explore prompting AI for more diverse or counter‐stereotypical perspectives or using this method to critique existing simulation scenarios. Our epistemological framing, blending interpretivism with AI generation, presents inherent interpretive tensions.

## 5 CONCLUSIONS

Patient mannequins, though silent, are central figures in the theatre of medical education. By playfully employing AI to give them a voice and then seriously analysing these fictional narratives, we sought to illuminate their pedagogical roles from an unconventional angle. This exploration serves as both a whimsical reflection on the mannequin ‘condition’ and a serious reminder of core principles in simulation‐based learning – communication, teamwork, empathy and navigating complexity. It highlights mannequins as vital, albeit non‐human, contributors to the development of future healthcare professionals. Moreover, the study demonstrates a novel methodological application of AI, prompting further dialogue about creative approaches to understanding and enhancing educational practices in an era of rapid technological change. Joking aside, perhaps listening to our ‘silent teachers’, even through the filter of AI, can offer valuable lessons.

## AUTHOR CONTRIBUTIONS

**Aaron Lawson McLean:** Conceptualization; investigation; writing–original draft; methodology; writing—review and editing; formal analysis. **Anna C. Lawson McLean:** Investigation; writing—review and editing; methodology; formal analysis.

## CONFLICT OF INTEREST STATEMENT

The authors declare no potential conflicts of interest with respect to the research, authorship and/or publication of this article.

## ETHICS STATEMENT

Formal ethical approval was not required according to applicable legislation and institutional guidance.

## AUTHORSHIP STATEMENT

In accordance with ICMJE criteria, both authors, ALM and ACLM, made substantial contributions to the work and meet the requirements for authorship. They have reviewed and approved the final submitted version and take full responsibility for its integrity and accuracy.

## PATIENT CONSENT FOR PUBLICATION

Not applicable.

## Supporting information


**Supplementary Material**: AI Prompting Process.

## Data Availability

The data that support the findings of this study are available from the corresponding author upon reasonable request.
