# Supplementary material for: Silent teachers: Narratives from the simulation lab
Source: Med Educ. 2025 May 20;60(1):46–50. doi: 10.1111/medu.15711 (PMC12717498; doi:10.1111/medu.15711)
Supplement: Supplementary file 1 — Supplementary Material: AI Prompting Process. [file MEDU-60-46-s001.docx]

**Supplementary Material: AI Prompting Process**

To generate the narratives, researchers engaged in an iterative, conversational prompting process with ChatGPT 4.0. The initial prompts aimed to establish the persona and context for each mannequin.

**Example Initial Prompt Structure (Adapted for Each Mannequin):**

Imagine you are a patient simulation mannequin named [Mannequin Name, e.g., Mr. Heart Attack]. You have been used for many years in a medical simulation lab to teach [Specific Skills/Scenarios, e.g., ACLS and resuscitation]. Describe your ‘life’ and ‘experiences’ from your first-person perspective. What do you 'see' and 'feel' during simulations? What are your ‘thoughts’ about the students who learn on you? What is your ‘purpose’? Write a narrative of about 500-700 words, adopting a [Suggested Tone, e.g., slightly world-weary but dedicated] tone.

**Follow-up Prompts:**

Based on the initial output, researchers used follow-up prompts to refine the narratives, encouraging deeper reflection or specific details. Examples include:

- Can you elaborate more on the teamwork aspect you ‘observe’?
- Tell me more about a particularly memorable or challenging simulation session from your ‘perspective’.
- How do you 'feel' about the debriefing sessions that happen after a simulation?
- Reflect on the emotional responses you ‘see’ in the learners.
- “Can you incorporate a specific example related to [e.g., polypharmacy management for Triplets / communication during delivery for Mrs. Difficult Birth]?”

This iterative process continued until the narratives reached a state deemed sufficiently rich and representative of the intended archetypal roles for the purpose of the subsequent qualitative analysis. The final narratives generated by the AI were longer than the excerpts presented in Table 1 of the main manuscript; the table provides illustrative snapshots.
